# Ethanol induces interferon expression in neurons via TRAIL: role of astrocyte-to-neuron signaling

**DOI:** 10.1007/s00213-018-5153-8

**Published:** 2019-01-04

**Authors:** Colleen J. Lawrimore, Leon G. Coleman, Fulton T. Crews

**Affiliations:** 1 Bowles Center for Alcohol Studies, School of Medicine, University of North Carolina at Chapel Hill, Chapel Hill, NC USA; 2 Curriculum in Neurobiology, University of North Carolina at Chapel Hill, Chapel Hill, NC USA; 3 Department of Pharmacology, University of North Carolina at Chapel Hill, Chapel Hill, NC USA; 4 Department of Psychiatry, University of North Carolina at Chapel Hill, Chapel Hill, NC USA

**Keywords:** Innate immune, Alcohol, Interferons, TRAIL, Astrocytes, Neurons

## Abstract

**Rationale:**

Alcohol use disorder (AUD) involves dysregulation of innate immune signaling in brain. Toll-like receptor 3 (TLR3), an innate immune receptor that is upregulated in post-mortem human alcoholics, leads to induction of interferon (IFN) signaling. IFNs have been linked to depressive-like symptoms and therefore may play a role in addiction pathology. Astrocyte-neuronal signaling may contribute to maladaptation of neuronal circuits.

**Objectives:**

In this manuscript, we examine ethanol (EtOH) induction of IFN signaling in neuronal, astrocyte, and microglial cell lines and assess astrocyte-neuronal interactions.

**Methods:**

U373 astrocytes, SH-SY5Y neurons, and BV2 microglia were treated with EtOH and analyzed for autocrine/paracrine IFN signaling.

**Results:**

EtOH induced TLR3, IFNβ, and IFNγ in SH-SY5Y neurons and U373 astrocytes, but not in BV2 microglia. The IFN response gene TRAIL was also strongly upregulated by TLR3 agonist Poly(I:C) and EtOH in U373 astrocytes. TRAIL blockage via neutralizing antibody prevented induction of IFNs in SH-SY5Y neurons but not in U373 astrocytes. Blocking TRAIL in conditioned media from EtOH-treated astrocytes prevented induction of IFNs in SH-SY5Y neurons. Finally, an in vivo model of chronic 10-day binge EtOH exposure in C57BL6/J mice, as well as single acute treatment with Poly(I:C), showed increased TRAIL +IR cells in both orbitofrontal and entorhinal cortex.

**Conclusions:**

This study establishes a role of astrocyte to neuron TRAIL release in EtOH-induced IFN responses. This may contribute to alcohol associated negative affect and suggest potential therapeutic benefit of TRAIL inhibition in AUD.

## Introduction

Alcohol use disorder (AUD) features prominent dysregulation of innate immune signaling in brain (Crews et al. 2017; Mayfield et al. 2013). AUD is also associated with progressively increasing negative-affect or depressive symptoms that are thought to promote the development and worsening of the disease (Boden and Fergusson 2011). Innate immune signaling, particularly interferons (IFNs), has been associated with the development of depression in humans and in vivo (Borsini et al. 2017; Callaghan et al. 2018; Fritz et al. 2018; Mina et al. 2015; Pinto and Andrade 2016). IFNγ was recently found to be upregulated in postmortem human alcoholic cortex (Johnson et al. 2015), and chronic ethanol (EtOH) induces IFNγ in vivo (Duncan et al. 2016; Pascual et al. 2015). Negative affect is a key aspect in the cycle of addiction, which is thought to drive self-administration (Koob 2015; Koob and Volkow 2010; Volkow et al. 2016). The induction of IFNs by EtOH, as well as the association of IFNs with negative affect, suggests a potential contribution for IFNs in the negative affective stage of addiction pathology. However, little is known about the mechanism of EtOH-induction of IFNs.

Toll-like receptors (TLRs), which are an important part of the innate immune system that respond to viral and bacterial components, also recognize endogenous agonists to promote sterile inflammation. TLRs are upregulated in post-mortem human alcoholic brain (for review, see Crews et al. 2017) as well as in rodent models (Lippai et al. 2013). Endosomal TLRs initiate IFN responses ultimately by induction of interferon regulatory factor (IRF) transcription factors. TLR3 is an endosomal TRIF-dependent TLR that results in IRF3 activation and IFN gene induction. TLR3 has been linked to alcohol drinking behavior in rodents (Jang et al. 2016), and 10-day binge EtOH treatment in mice not only upregulates TLR3 but also potentiates cytokine responses following systemic treatment with TLR3 agonist Poly(I:C) (Qin and Crews 2012). TLR3 is induced by EtOH in the prefrontal cortex and nucleus accumbens following alcohol self-administration (McCarthy et al. 2017b). We have shown previously that TLR3 is upregulated in postmortem human alcoholic brain, as well as in SH-SY5Y neurons in vitro (Lawrimore and Crews 2017). Thus, it is critical to understand the role of TLR3 and IFN induction in response to EtOH.

In peripheral macrophages, it has been well established that TLR3 activation leads to induction of type I (e.g., IFNβ) and type II (IFNγ) IFNs (Sen and Sarkar 2005) as well as a host of interferon response genes (IRGs) (Levy and Darnell Jr 2002). These include antiviral proteins, such as TNF-related apoptosis-inducing ligand (TRAIL/TNFSF10/APO2L/CD253). TRAIL can cause either cell death in TRAIL cell death-sensitive cells or IFN and other cytokine induction. This has been observed in multiple cell types (Croft and Siegel 2017; Henry and Martin 2017; Kumar-Sinha et al. 2002; Wilson et al. 2009). For instance, TRAIL does not induce cell death in T cells (LeBlanc and Ashkenazi 2003), though neuronal lines can be sensitized to TRAIL-mediated cell death by NGF (Ruggeri et al. 2016). However, IFN and TRAIL signaling, as well as how EtOH impacts these pathways, is still poorly understood among different brain cell types. We hypothesized that TRAIL signaling would regulate IFN induction in neurons.

Multiple studies have reported that EtOH sensitizes microglia, inducing a hyper-ramified morphology associated with increases in cytokine secretion. Although microglia are brain-specific innate immune monocytes, we have previously found that neurons also play a role in EtOH-induced innate immune signaling that is unique from that of microglia (Lawrimore and Crews 2017). Astrocytes also have immunological properties and have been shown to play an important role in EtOH-induced inflammation (Adermark and Bowers 2016), although the effect of EtOH is poorly understood. Innate immune signaling in brain shares the autocrine (cell-to-self signaling) and paracrine (cell-to-cell signaling) mechanisms found among systemic innate immune cells. For example, neuronal ligand CX3CL1 (fractalkine) signals to microglia through CX3CR1 altering immune signaling, synaptic plasticity (Sheridan et al. 2014), as well as amyloid β-complement processing in Alzheimer’s disease models (Lian et al. 2015). Although it is known that EtOH can alter astrocyte activation, little is known about astrocyte innate immune signaling and how it might impact neurons through paracrine signaling pathways in brain.

In this manuscript, we utilized neuronal, microglial, and astrocytic cell lines (SH-SY5Y, BV2, and U373, respectively) to examine EtOH-induced IFN signaling in each cell type. These cell lines have been used extensively in the study of their respective cell type (Cheung et al. 2009; Henn et al. 2009; Imaizumi et al. 2013; Korecka et al. 2013; Rosenberger et al. 2016) and importantly, enabled us to conduct experiments without possibility of contamination of other cell types that is common in primary cultures. We also examine astrocyte to neuronal signaling in the context of neuronal IFN induction. Interestingly, although BV2 microglia do respond to EtOH (Lawrimore and Crews 2017), the response did not include induction of TLR3 or IFNs. In contrast, in SH-5Y5Y neurons and U373 astrocytes, EtOH treatment resulted in a strong induction of TLR3, IFNβ, and IFNγ in both cell types. TRAIL was also strongly upregulated by both EtOH and TLR3 agonist Poly(I:C) in U373 astrocytes. TRAIL neutralizing antibody prevented EtOH induction of IFN in SH-SY5Y neurons, suggesting that TRAIL released by EtOH stimulates autocrine induction of IFN in neurons. Similarly, TRAIL neutralizing antibody blocked IFN induction in neurons by EtOH-conditioned U373-astrocyte media consistent with astrocyte TRAIL-mediated paracrine induction of IFN in neurons. Furthermore, mice treated systemically with TLR3 agonist Poly(I:C) or EtOH show increases in cortical TRAIL positive immunoreactive cells. These studies link EtOH to novel IFN-TRAIL innate immune signaling between neurons and astrocytes, which may contribute to the pathology of AUD.

## Materials and methods

### Cell lines and treatment reagents

BV2 microglia cells were acquired from ICLC (Genoa, Italy, #ATL03001). BV2 were cultured using Dubecco’s modified Eagle serum (DMEM, Life Technologies, Carlsbad, CA) supplemented with 10% fetal bovine serum (FBS, Life Technologies), 1× GlutaMAX (Life Technologies), and 1× antibiotic-antimycotic (Life Technologies). For all cell experiments (excluding MTT assay), polystyrene 6-well plates (Corning, Corning, NY, #3516) containing a culture area of 9.5 cm^2^ per well were used. BV2 were plated at 1.5 × 10^5 cells per well, and 16 h prior to treatments, media was aspirated, and then 2% FBS was added.

SH-SY5Y neuroblastoma cells were acquired from ATCC (Manassas, VA, #CRL-2266). SH-SY5Y were cultured using DMEM/F-12 + GlutaMAX (Life Technologies), 10% FBS, and 1× antibiotic-antimycotic. Prior to treatments, SH-SY5Y were plated in 6-well plates (3.75 × 10^5 per well), and 24 h after plating, cells were differentiated using 10-μM retinoic acid (RA, Sigma-Aldrich, St. Louis, MO, #R2625) for 4 days in Neurobasal media (Life Technologies) containing 2% B27 supplement (Life Technologies), 0.5-mM GlutaMAX, and 1× antibiotic-antimycotic, which induces a more mature neuronal phenotype (Kovalevich and Langford 2013). Media were refreshed 16 h prior to treatments.

U373-MG (U373) astroglioma cells were acquired from UNC Cell Culture Facility. U373 were cultured using Alpha MEM (Life Technologies), 10% FBS, and 1× antibiotic-antimycotic. U373 were plated in 6-well plates at 3.25 × 10^5 cells per well, and then 16 h prior to treatments, media was changed to 2% FBS.

All cell types were maintained in a humidified 5% CO_2_ incubator at 37 °C. Cells were treated at 90% confluency. Reagents used included EtOH (timepoint and concentration dependent on experiment; see results), Poly(I:C) (50 μg/mL, Amersham/GE Healthcare, Pittsburgh, PA, #27-4732-01), LPS (100 ng/mL, Sigma-Aldrich, #L2630), Imiquimod (1 μg/mL, Invivogen, #tlrl-imq), TRAIL (200 ng/mL, Millipore, #GF092), and TRAIL neutralizing antibody (2 μg/mL, BD Biosciences, #550912).

### Conditioned media transfer

SH-SY5Y were cultured and differentiated with retinoic-acid as described in the previous section. U373 were cultured then treated with EtOH (100 mM, 24 h). Following treatment, media were collected, transferred to empty 6-well plates, and the EtOH was allowed to evaporate overnight as previously described (Walter and Crews 2017). Media were removed from SH-SY5Y, and the conditioned media from U373 were then transferred to each respective group. Immediately following media transfer, the TRAIL neutralizing antibody (2 μg/mL) was added to the appropriate groups. Cell lysates were harvested 24 h later for mRNA analysis using RT-PCR.

### Mouse treatment

Male C57BL/6 mice (8 weeks old) were treated with either EtOH [5 g/kg, 25% *w*/*v*, intragastric (i.g.)] or water (i.g.) daily for 10 days, or were treated with a single dose of LPS [0.5 mg/kg, intraperitoneal (i.p.)], Poly(I:C) (13 mg/kg, i.p.), or saline (i.p.) as previously described (Qin and Crews 2012; Qin et al. 2008). Mice were sacrificed 24 h following the last dose. Following each treatment, mice were anesthetized using sodium pentobarbital, then transcardially perfused with 0.1-M PBS, followed by 4.0% paraformaldehyde in PBS. Brains were removed and sent to Neuroscience Associates (Knoxville, TN) for histological sectioning. Mice were housed in a temperature- (20 °C) and humidity-controlled vivarium on a 12-h/12-h light/dark cycle and provided ad libitum access to food and water. Experimental procedures were approved by the Institutional Animal Care and Use Committee of the University of North Carolina at Chapel Hill and conducted in accordance with National Institutes of Health regulations for the care and use of animals in research.

### Real-time PCR

Following treatments, cells were rinsed using ice cold PBS, then lysed using TRIzol (Invitrogen, Carlsbad, CA). Cells were homogenized using a microtube homogenizer system (Wilmad-Labglass, Vineland, NJ). Tubes were allowed to sit at room temperature (RT) for 5 min, then chloroform was added to each tube followed by mixing on table rocker for 10 min at RT. Tubes were spun at 13,800 ×*g* for 15 min at 4 °C, and the aqueous (RNA containing) phase was transferred to a new tube. An equal volume of 100% isopropranol was added to each tube, followed by mixing on a table rocker for 10 min, then spun at 13,800 ×*g* for 15 min at 4 °C. The RNA pellet was rinsed twice with cold 75% EtOH, allowed to air dry for 10 min at RT, then re-suspended in DEPEC-treated water. RNA concentration was determined using a Nanodrop (Thermo Scientific, Waltham, MA) and was reverse-transcribed to cDNA. The SYBR green PCR master mix (Life Technologies) was used for real-time (RT) PCR analysis. The relative differences in expression between groups were expressed using cycle time (Ct) values normalized with β-actin, and relative differences between control and treatment groups were calculated and expressed as relative increases setting control as 100%. Primers used are listed in Table 1.

**Table 1:** Primer sequences for RT-PCR

Gene	Species	Forward (5′–3′)	Reverse (5–3′)
Caspase 8	Human	GGCACAGAGATTAAGTCCATTGAT	GGACACACAGACTCGAATGC
CD14	Human	AGAGGCAGCCGAAGAGTTCAC	GCGCTCCATGGTCGATAAGT
DR3	Human	AGAGATACTGACTGTGGGACC	CCCAGAACACACCTACTCTGC
DR4	Human	ACCTTCAAGTTTGTCGTCGTC	CCAAAGGGCTATGTTCCCATT
DR5	Human	GCCCCACAACAAAAGAGGTC	AGGTCATTCCAGTGAGTGCTA
FADD	Human	GAAAACGCGCTCTTGTCGAT	GCCCGAGGCATAGGAACTTG
Fas	Human	GTCTCCTGCGATGTTTGGC	TTCAAGGAAAGCTGATACCTATTTC
HMGB1	Human	GGAGATCCTAAGAAGCCGAGA	CATGGTCTTCCACCTCTCTGA
IFNAR1	Human	ATTTACACCATTTCGCAAAGCTC	TCCAAAGCCCACATAACACTATC
IFNAR2	Human	TCATGGTGTATATCAGCCTCGT	AGTTGGTACAATGGAGTGGTTTT
IFNβ	Human	CACAACAGGTAGTAGGCGACA	AGAAGCACAACAGGAGAGCA
IFNγ	Human	TCAGCTCTGCATCGTTTTGG	GTTCCATTATCCGCTACATCTGAA
IFNγR1	Human	TCTTTGGGTCAGAGTTAAAGCCA	TTCCATCTCGGCATACAGCAA
IFNγR2	Human	TCACCGTCCTAGAAGGATTCAG	AAACTCTGGTGGTTCAAAAGACA
IKKβ	Human	CTGGCCTTTGAGTGCATCAC	CGCTAACAACAATGTCCACCT
IL-10	Human	GACTTTAAGGGTTACCTGGGTTG	TCACATGCGCCTTGATGTCTG
IL-1β	Human	ATGATGGCTTATTACAGTGGCAA	GTCGGAGATTCGTAGCTGGA
IL-6	Human	ACTCACCTCTTCAGAACGAATTG	CCATCTTTGGAAGGTTCAGGTTG
MCP1	Human	CTCTCGCCTCCAGCATGAAA	AGGGTGTCTGGGGAAAGCTA
MD-2	Human	GAAGCTCAGAAGCAGTATTGGGTC	GGTTGGTGTAGGATGACAAACTCC
NGFR	Human	CCTACGGCTACTACCAGGATG	CACACGGTGTTCTGCTTGT
RAGE	Human	CTACCGAGTCCGTGTCTACCA	CATCCAAGTGCCAGCTAAGAG
S100B	Human	CTTTCCAGCCGTGTTGTAGC	CTGCATGGATGAGGAACGCA
TLR3	Human	TTGCCTTGTATCTACTTTTGGGG	TCAACACTGTTATGTTTGTGGGT
TLR4	Human	CTCTGGGGAGGCACATCTTC	CCCAGGTGAGCTGTAGCATT
TLR7	Human	GATAACAATGTCACAGCCGTCC	GTTCCTGGAGTTTGTTGATGTTC
TNFα	Human	CCCAGGCAGTCAGATCATCTTCT	ATGAGGTACAGGCCCTCTGAT
TRAIL	Human	TGCGTGCTGATCGTGATCTTC	GCTCGTTGGTAAAGTACACGTA
β-Actin	Human	GATGCAGAAGGAGATCACTGC	ATACTCCTGCTTGCTGATCCA
IFNβ	Mouse	AGCTCCAAGAAAGGACGAACA	GCCCTGTAGGTGAGGTTGAT
IFNγ	Mouse	ATGAACGCTACACACTGCATC	CCATCCTTTTGCCAGTTCCTC
TLR3	Mouse	GTGAGATACAACGTAGCTGACTG	TCCTGCATCCAAGATAGCAAGT
β-Actin	Mouse	GTATGACTCCACTCACGGCAAA	GGTCTCGCTCCTGGAAGATG

### Enzyme-linked immunosorbent assays (ELISAs)

Following treatments, 1 mL of media was collected and spun down at 500 ×*g* for 10 min to remove cell debris. Supernatant was transferred to a new tube and stored at − 20 until analysis. Media were analyzed using human TRAIL/TNFSF10 DuoSet ELISA (R&D Systems, #DY375) per the manufacturer’s instructions using undiluted media samples.

### MTT assay

SH-SY5Y were plated on a polystyrene 96 well plate (Corning, #3596) at 1 × 10^5 cells per well and differentiated using retinoic acid as described in the previous section. Cells were treated with varying concentrations of TRAIL (1–1000 ng/mL) as well as TRAIL neutralizing antibody (2 μg/mL) that has been demonstrated to block TRAIL-induced apoptosis (Cantarella et al. 2003) in the appropriate groups in triplicate. Twenty-four hours following treatment, the MTT assay (Abcam, #ab211091) was used to assess cell death per the manufacturer’s instructions.

### Immunohistochemistry

Neuroscience Associates sectioned brains into coronal slices (35 μm) and were returned to the lab for immunohistochemical staining as previously described (Vetreno and Crews 2012; Vetreno et al. 2013). Briefly, sections were washed with PBS followed by a hydrogen peroxide (0.6%) rinse, then antigen retrieval using Citra solution at 70 °C for 1 h. Sections were then blocked using rabbit serum, followed by an overnight incubation in goat anti-TRAIL (1:50, R&D #AF1121). The next day, sections were incubated with rabbit anti-goat IgG (1:200) at RT for 1 h. Sections were then incubated in ABC solution at RT for 1 h, followed by visualization using nickel-enhanced 3,3′-diaminobenzidinne (DAB). Sections were quantified for immunopositive cells using Nikon software, setting threshold for cell size and intensity that was applied unbiased to all sections.

### Statistical analysis

Where appropriate, *t* tests or one-way ANOVAs with post hoc Tukey’s/Dunnet’s test were used. The particular test used for each data set is indicated in the appropriate figure legends. A *p* value of less than 0.05 was considered significant. Data is reported as mean ± standard error of the mean (SEM). All data analysis was conducted using Prism (Graphpad, La Jolla, CA).

## Results

### EtOH induces TLR3 and IFNs in U373 astrocytes and SH-SY5Y neurons, but not BV2 microglia

**Fig. 1:**
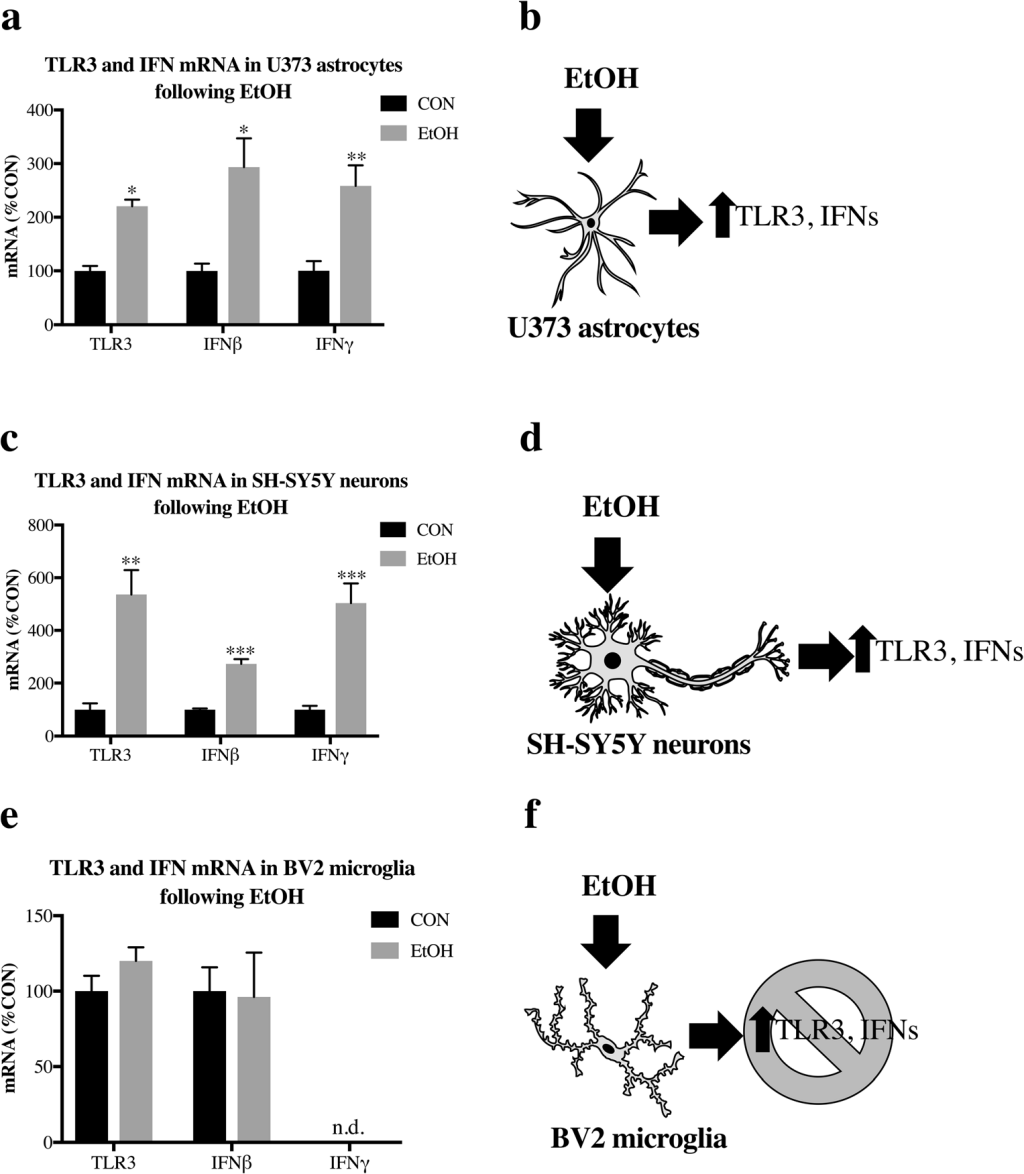
Ethanol increases TLR3, IFNs in U373 astrocytes and SH-SY5Y neurons, but not BV2 microglia. U373 astrocytes, SH-SY5Y neurons, and BV2 microglia were treated with EtOH (100 mM) for 24 h. Cell lysates were collected, and mRNA expression was measured using RT-PCR. Data is expressed as %CON (mean ± SEM), followed by analysis using two-tailed *t* tests. **a**, **b** EtOH increased expression of TLR3 (221 ± 12%), IFNβ (293 ± 54%), and IFNγ (258 ± 38%) in U373. **c**, **d** EtOH increased expression of TLR3 (536 ± 93%), IFNβ (273 ± 18%), and IFNγ (504 ± 75%) in SH-SY5Y. **e**, **f** EtOH did not increase either TLR3, IFNβ, or IFNγ in BV2. *n* = 5–6 per group; *p < 0.05, **p < 0.01, ***p < 0.001 vs. CON

**Fig. 2:**
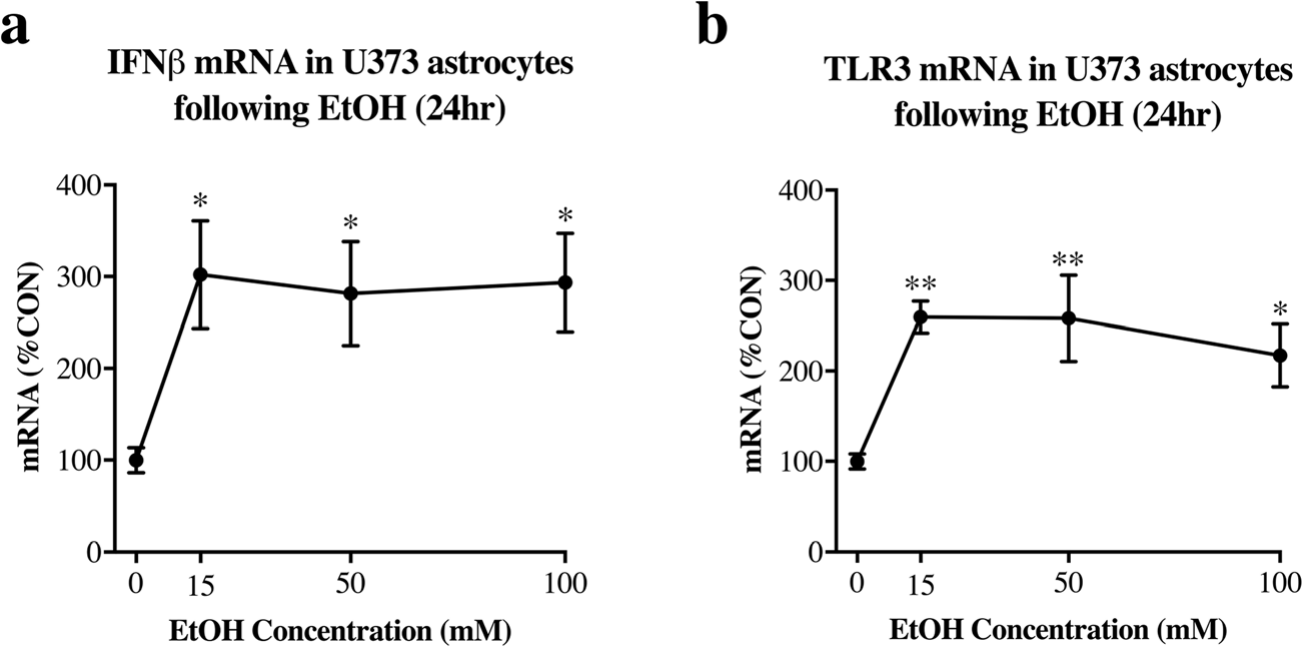
Ethanol increases IFNβ and TLR3 at low concentrations in U373 astrocytes. U373 astrocytes were treated with varying concentrations of EtOH (15, 50, or 100 mM) for 24 h. Cell lysates were collected, and mRNA expression was measured using RT-PCR. Data is expressed as %CON (mean ± SEM). **a** EtOH increased expression of IFNβ mRNA at 15 (302 ± 59%), 50 (282 ± 57%), and 100 mM (293 ± 54%). One-way ANOVA [*F*(3,17) = 3.52, *p* = 0.0379] followed by Dunnet’s post hoc. **b** EtOH increased expression of TLR3 mRNA at 15 (259 ± 18%), 50 (258 ± 48%), and 100 mM (217 ± 35%). One-way ANOVA [*F*(3,17) = 5.84, *p* = 0.0068] followed by Dunnet’s post hoc. *p < 0.05, **p < 0.01 vs. CON

Previous studies have found EtOH increases expression of TLR3 in mouse (Qin and Crews 2012), rats (Vetreno and Crews 2012), as well as in post-mortem human alcoholic brain (Crews et al. 2013). Furthermore, EtOH upregulates TLR3 in SH-SY5Y neurons, but not in BV2 microglia (Lawrimore and Crews 2017). To determine EtOH-induced cell type-specific responses in this pathway, expression of TLR3, IFNβ, and IFNγ was examined following EtOH treatment in U373 astrocytes, SH-SY5Y neurons, and BV2 microglia. EtOH increased expression of TLR3 (2.2-fold, *p* < 0.05), IFNβ (2.9-fold, *p* < 0.05), and IFNγ (2.6-fold, *p* < 0.01) in U373 astrocytes (Fig. 1a, b). Furthermore, in SH-SY5Y, EtOH increased expression of TLR3 (5.4-fold, *p* < 0.01), IFNβ (2.7-fold, *p* < 0.001), and IFNγ (5-fold, *p* < 0.001) (Fig. 1c, d). However, BV2 microglia did not show induction of TLR3 or IFNs in response to EtOH (Fig. 1e, f). U373 astrocytes were found to be quite sensitive to the effects of EtOH. We found that 15 mM of EtOH, which is below the legal driving limit in the USA, upregulated IFNβ (3-fold, *p* < 0.05) and TLR3 (2.6-fold, *p* < 0.01) at 24 h (Fig. 2). Furthermore, multiple IFN signaling genes, cytokines, and TNF superfamily genes were upregulated in a concentration and time-dependent manner in U373 astrocytes (see Tables 2 and 3). Also, we found that multiple markers of “A1” astrocyte activation (Liddelow et al. 2017), such as AMIGO2, GFAP, and FKBP5 were all upregulated by EtOH (100 mM, 24 h; data not shown). In SH-SY5Y, we further found that EtOH upregulated IFN receptors and multiple components of the TNF superfamily signaling genes (Table 4). Thus, EtOH induces TLR3 and IFN expression in U373 astrocytes and SH-SY5Y neurons, but not BV2 microglia.

**Table 2:** Summary of ethanol concentration curve in U373 astrocyte

U373 astrocyte EtOH concentration curve (24 h)				
Gene ID	CON	15 mM	50 mM	100 mM
IFN signaling genes				
IFNAR1	100 ± 16	149 ± 14	192 ± 51	117 ± 18
IFNAR2	100 ± 14	157 ± 19	191 ± 30	158 ± 16*
IFNβ	100 ± 14	302 ± 59*	282 ± 57*	293 ± 54*
IFNγ	100 ± 18	75 ± 6.7	156 ± 48	258 ± 38**
IFNγR1	100 ± 12	170 ± 16*	178 ± 31*	111 ± 14
IFNγR2	100 ± 7.9	165 ± 14*	154 ± 23*	137 ± 11
NGFR	100 ± 6.9	759 ± 134**	996 ± 124***	2663 ± 193****
TRAIL	100 ± 7.7	173 ± 16*	207 ± 40**	166 ± 22*
Cytokines and TLR signaling genes				
CD14	100 ± 9.5	143 ± 11	133 ± 22	94 ± 14
HMGB1	100 ± 16	122 ± 14	118 ± 25	88 ± 11
IL-10	100 ± 13	136 ± 9.8	101 ± 23	131 ± 23
IL-1β	100 ± 10	172 ± 18*	136 ± 22	162 ± 17*
IL-6	100 ± 5.8	140 ± 10	146 ± 18*	149 ± 11*
MCP1	100 ± 8.4	100 ± 7.5	89 ± 8.7	79 ± 3.3
MD2	100 ± 8.5	135 ± 6.7	127 ± 15	138 ± 14
RAGE	100 ± 15	209 ± 22**	191 ± 27*	163 ± 23
S100B	100 ± 12	115 ± 12	94 ± 13	74 ± 7.5
TLR3	100 ± 8.5	259 ± 18**	258 ± 48**	217 ± 35*
TLR4	100 ± 15	156 ± 28	171 ± 44	122 ± 17
TLR7	100 ± 11	155 ± 38	168 ± 46	138 ± 32
TNFα	100 ± 12	100 ± 30	107 ± 28	132 ± 10
TNF superfamily signaling genes				
Caspase 8	100 ± 30	124 ± 34	144 ± 35	91 ± 19
TNFRSF25 (DR3)	100 ± 9.8	166 ± 8.9**	170 ± 14**	167 ± 14**
TNFRSF10A (DR4)	100 ± 11	93 ± 22	144 ± 23	201 ± 31*
TNFRSF10B (DR5)	100 ± 6.8	102 ± 6.3	119 ± 19	108 ± 9.8
FADD	100 ± 6.0	118 ± 3.0*	114 ± 2.9	125 ± 5.8**
Fas	100 ± 29	108 ± 30	130 ± 39	65 ± 12

U373 astrocytes were treated with EtOH at varying concentrations for 24 h. Cell lysates were collected for mRNA analysis using RT-PCR. Data is expressed as %CON. *n* = 5–6 per group. Significantly changed genes are indicated in bold

*p < 0.05

**p < 0.01

***p < 0.001

****p < 0.0001 vs. CON

**Table 3:** Summary of ethanol time course in U373 astrocytes

U373 astrocyte EtOH timecourse (15 mM)				
Gene ID	0 h	1 h	6 h	24 h
IFN signaling genes				
IFNβ	100 ± 20	120 ± 44	67 ± 12	269 ± 60*
IFNγ	100 ± 17	142 ± 29	122 ± 39	133 ± 22
IFNγR1	100 ± 13	138 ± 27	132 ± 33	328 ± 73**
IFNγR2	100 ± 14	105 ± 16	119 ± 28	226 ± 43*
TRAIL	100 ± 12	157 ± 31	169 ± 31	470 ± 118***
Cytokines and receptors				
IL-1β	100 ± 12	128 ± 23	187 ± 50	447 ± 98***
IL-6	100 ± 11	108 ± 16	84 ± 13	171 ± 30*
RAGE	100 ± 17	139 ± 29	115 ± 17	324 ± 60***

U373 astrocytes were treated with EtOH (15 mM) at varying timepoints. Cell lysates were collected for mRNA analysis using RT-PCR. Data is expressed as %CON. *n* = 5–6 per group. Significantly changed genes are indicated in bold

*p < 0.05

**p < 0.01

***p < 0.001 vs. CON

**Table 4:** Summary of ethanol effects on IFN and TNF superfamily in SH-SY5 neurons

SH-SY5Y neuron EtOH treatment		
Gene ID	CON	EtOH
IFN signaling genes		
IFNβ	100 ± 5.1	273 ± 18***
IFNγ	100 ± 15	504 ± 75***
IFNAR1	100 ± 9.7	345 ± 42***
IFNAR2	100 ± 8.5	297 ± 21****
IFNγR1	100 ± 15	461 ± 84**
IFNγR2	100 ± 12	328 ± 29****
NGFR	100 ± 12	5127 ± 471****
TRAIL	100 ± 12	432 ± 92**
TNF superfamily signaling genes		
Caspase 8	100 ± 21	251 ± 30**
TNFRSF25 (DR3)	100 ± 33	178 ± 33
TNFRSF10A (DR4)	100 ± 11	164 ± 12**
TNFRSF10B (DR5)	100 ± 9.7	137 ± 7.5*
FADD	100 ± 7.1	124 ± 12

SH-SY5Y neurons were treated with EtOH (100 mM, 24 h). Cell lysates were collected for mRNA analysis using RT-PCR. Data is expressed as %CON. *n* = 5–6 per group. Significantly changed genes are indicated in bold

*p < 0.05

**p < 0.01

***p < 0.001

****p < 0.0001 vs. CON

### Stimulation with TLR3 agonist Poly(I:C) increases IFN expression and IFN response gene TRAIL in U373 astrocytes

**Fig. 3:**
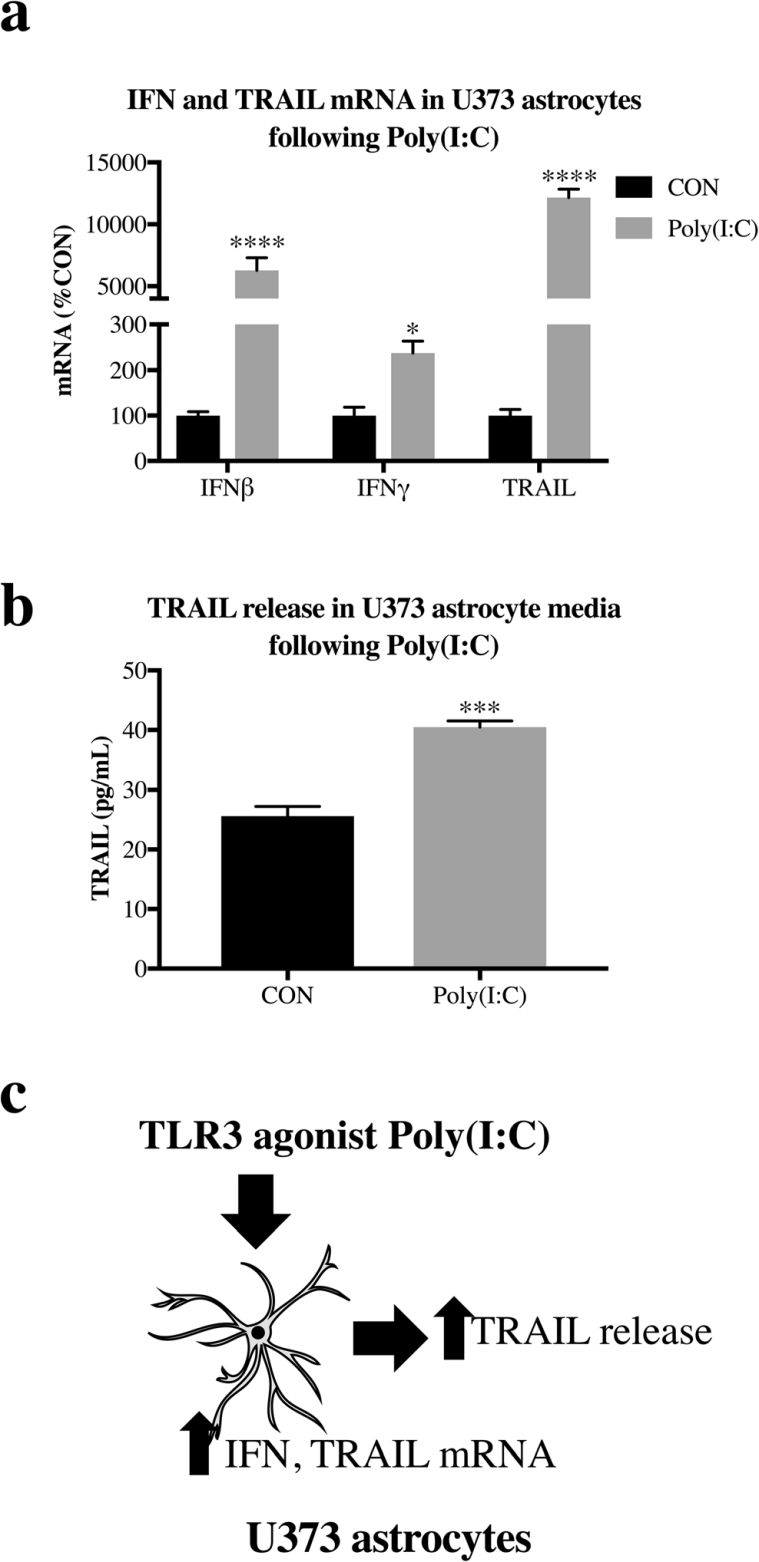
Stimulation with TLR3 agonist Poly(I:C) increases IFN expression and IFN response gene TRAIL in U373 astrocytes. U373 astrocytes were treated with TLR3 agonist Poly(I:C) (50 μg/mL) for 24 h. Cell lysates were collected for mRNA analysis using RT-PCR, with data expressed as %CON (mean ± SEM). Cell media was collected for analysis of released TRAIL using ELISA. Statistical analysis conducted using two-tailed t-tests. **a** Poly(I:C) increased expression of IFNβ (6283 ± 1008%), IFNγ (238 ± 26%), and TRAIL (12,151 ± 689%). **b** Poly(I:C) increased release of TRAIL (158 ± 4.1%) in the media. **c** Schematic summarizing Poly(I:C) effects on IFN/TRAIL signaling in U373 astrocytes. *n* = 5–6 per group; ***p < 0.001, ****p < 0.0001 vs. CON

Astrocytes show clear induction of TLR3 and IFNs in response to EtOH. We previously assessed the impact of TLR3 activation in SH-SY5Y neurons and BV2 microglia (Lawrimore and Crews 2017). In order to now investigate TLR3 signaling in U373 astrocytes, cells were treated with TLR3 agonist Poly(I:C) (50 μg/mL, 24 h). Poly(I:C) strongly upregulated both IFNβ (63-fold, *p* < 0.0001), IFNγ (2.4-fold, *p* < 0.05), and IFN regulated gene TNFSF10, also known as TRAIL (121-fold, *p* < 0.0001) (Fig. 3a). Furthermore, Poly(I:C) increased TRAIL secretion into the media (1.6-fold, *p* < 0.001) (Fig. 3b). In contrast, stimulation of TLR4 with LPS showed weaker responses, and stimulation of TLR7 with imiquimod showed no significant induction in the immune genes assessed (see Table 5). The concentrations of LPS (Shin et al. 2014) and imiquimod (Gay et al. 2013) tested cause significant immune gene induction in microglia. Thus, U373 astrocytes show induction of IFNs and TRAIL in response to TLR3.

**Table 5:** Summary of TLR3 agonist Poly(I:C), TLR4 agonist LPS, and TLR7 agonist imiquimod treatment in U373 astrocytes

U373 astrocyte TLR agonist treatment (24 h)				
Gene ID	CON	Poly(I:C)	LPS	Imiquimod
IFN signaling genes				
IFNAR1	100 ± 22	71 ± 5.2	51 ± 16	72 ± 21
IFNAR2	100 ± 10	174 ± 5.5****	58 ± 7.5**	68 ± 5.1*
IFNβ	100 ± 8.9	6284 ± 1008***	236 ± 16*	138 ± 24
IFNγ	100 ± 18	238 ± 26*	142 ± 45	79 ± 20
IFNγR1	100 ± 18	367 ± 32****	72 ± 18	83 ± 17
IFNγR2	100 ± 12	169 ± 4.5****	56 ± 6.9**	69 ± 6.2*
NGFR	100 ± 12	96 ± 8.4	79 ± 11	88 ± 11
TRAIL	100 ± 14	12,152 ± 689****	345 ± 64**	81 ± 14
Cytokines and TLR signaling genes				
IKKβ	100 ± 6.9	130 ± 6.1	86 ± 11.3	87 ± 9.8
IL-1β	100 ± 11.5	2911 ± 133****	252 ± 24***	103 ± 14
IL-6	100 ± 7.0	63,999 ± 3037****	150 ± 14*	89 ± 9.1
MCP1	100 ± 21	28,297 ± 800****	1241 ± 81****	156 ± 41
TLR3	100 ± 22	2839 ± 307****	401 ± 106*	134 ± 41
TLR7	100 ± 11	118 ± 7.0	71 ± 13	81 ± 14
TNFα	100 ± 8.7	28,331 ± 1864****	583 ± 72****	93 ± 10

U373 astrocytes were treated with TLR3 agonist Poly(I:C) (50 μg/mL), TLR4 agonist LPS (100 ng/mL), or TLR7 agonist imiquimod (1 μg/mL) for 24 h. Cell lysates were collected for mRNA analysis using RT-PCR. Data is expressed as %CON. *n* = 5–6 per group. Significantly changed genes are indicated in bold

*p < 0.05

**p < 0.01

***p < 0.001

****p < 0.0001 vs. CON

### EtOH increases TRAIL expression and release in U373 astrocytes

**Fig. 4:**
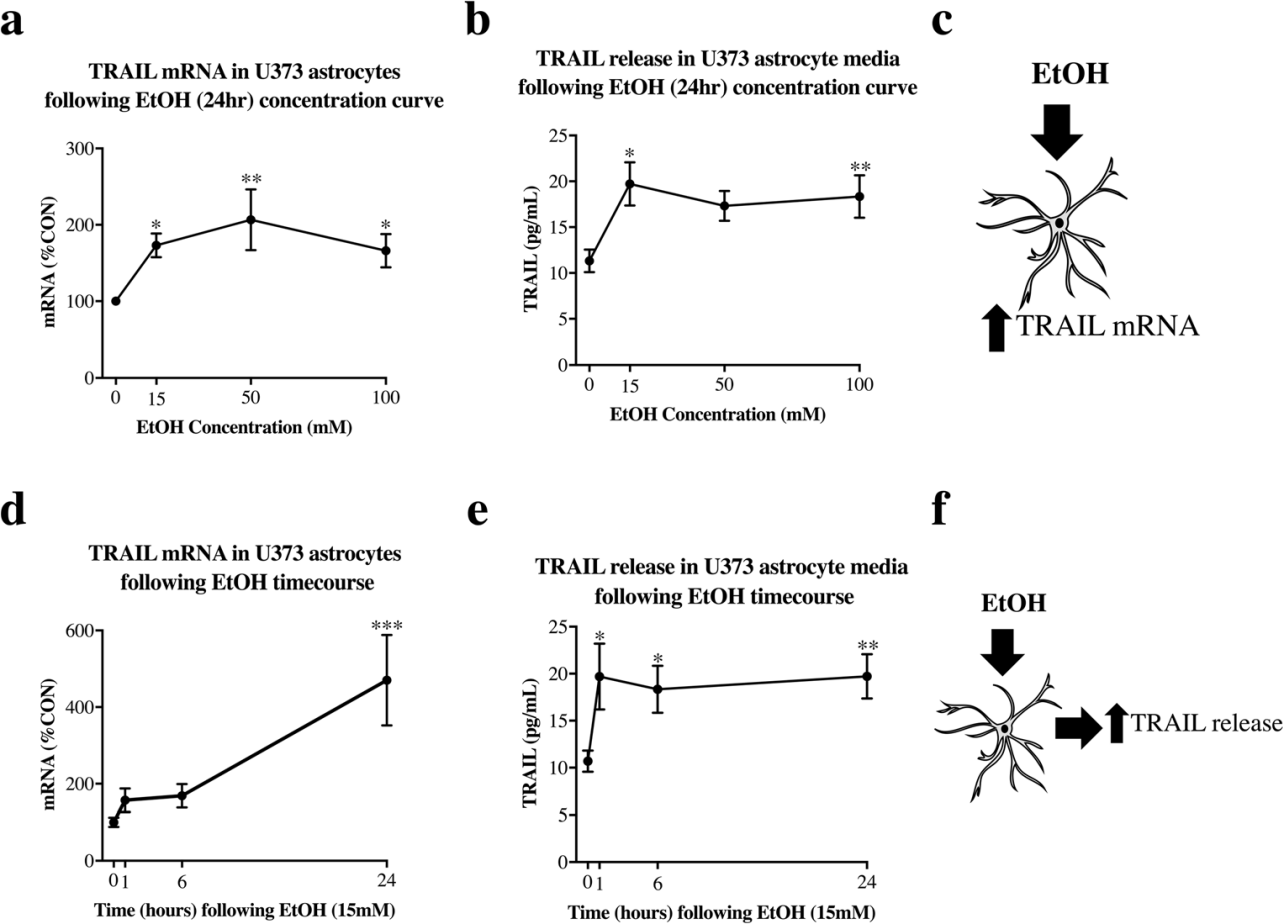
Ethanol increases TRAIL expression and release in U373 astrocytes. U373 astrocytes were treated with EtOH at varying concentrations and timepoints. Cell lysates were collected for mRNA analysis using RT-PCR, and cell media was collected for analysis of released TRAIL using an ELISA. RT-PCR data is expressed as %CON (mean ± SEM). **a** EtOH upregulated TRAIL mRNA at 15 (173 ± 17%), 50 (207 ± 41%), and 100 mM (166 ± 23%). One-way ANOVA [*F*(3,29) = 5.0, *p* = 0.0065] followed by Dunnet’s post hoc. **b** EtOH increased TRAIL release in the media at 15 (174 ± 28%) and 100 mM (162 ± 27%). One-way ANOVA [*F*(3,38) = 5.21, *p* = 0.0041] followed by Dunnet’s post hoc. **c** Schematic illustrating EtOH increases TRAIL mRNA in U373 astrocytes. **d** EtOH increased TRAIL mRNA at 24 (469 ± 132%) h. One-way ANOVA [*F*(3,18) = 7.88, *p* = 0.0014] followed by Dunnet’s post hoc. **e** EtOH increased TRAIL release in the media at 1 (174 ± 36%), 6 (162 ± 28%), and 24 (174 ± 28%) h. One-way ANOVA [*F*(3,35) = 7.32, *p* = 0.0006] followed by Dunnet’s post hoc. **f** Schematic illustrating EtOH increases TRAIL release in U373 astrocytes. *p < 0.05, **p < 0.01, ***p < 0.001 vs. CON

TRAIL is a type II transmembrane protein that is cleaved and released where it acts on multiple receptors. TRAIL is linked to IFN signaling, being both induced by IFNs as well as induction of IFNs, dependent upon cell type (Kumar-Sinha et al. 2002). Since we found that EtOH increases TLR3 in U373 and that TLR3 stimulation increases TRAIL in U373, we next examined whether EtOH could also induce TRAIL in U373 astrocytes. We treated U373 astrocytes with EtOH and examined both TRAIL mRNA and release in the media. We found that EtOH increased TRAIL mRNA expression at 15 mM (1.7-fold *p* < 0.05), 50 mM (2-fold, *p* < 0.01), and 100 mM (1.7-fold, *p* < 0.05) (Fig. 4a, as well as released TRAIL as low as 15 mM (1.7-fold, *p* < 0.05) (Fig. 4b). EtOH rapidly released TRAIL protein into the media (Fig. 4e) that was accompanied by a delayed slow increase in mRNA even at 15 mM (Fig. 4d). Thus, EtOH promotes induction of TRAIL release and expression in U373 astrocytes.

### EtOH induces IFNs in human SH-SY5Y neurons via TRAIL, but not in U373 astrocytes

**Fig. 5:**
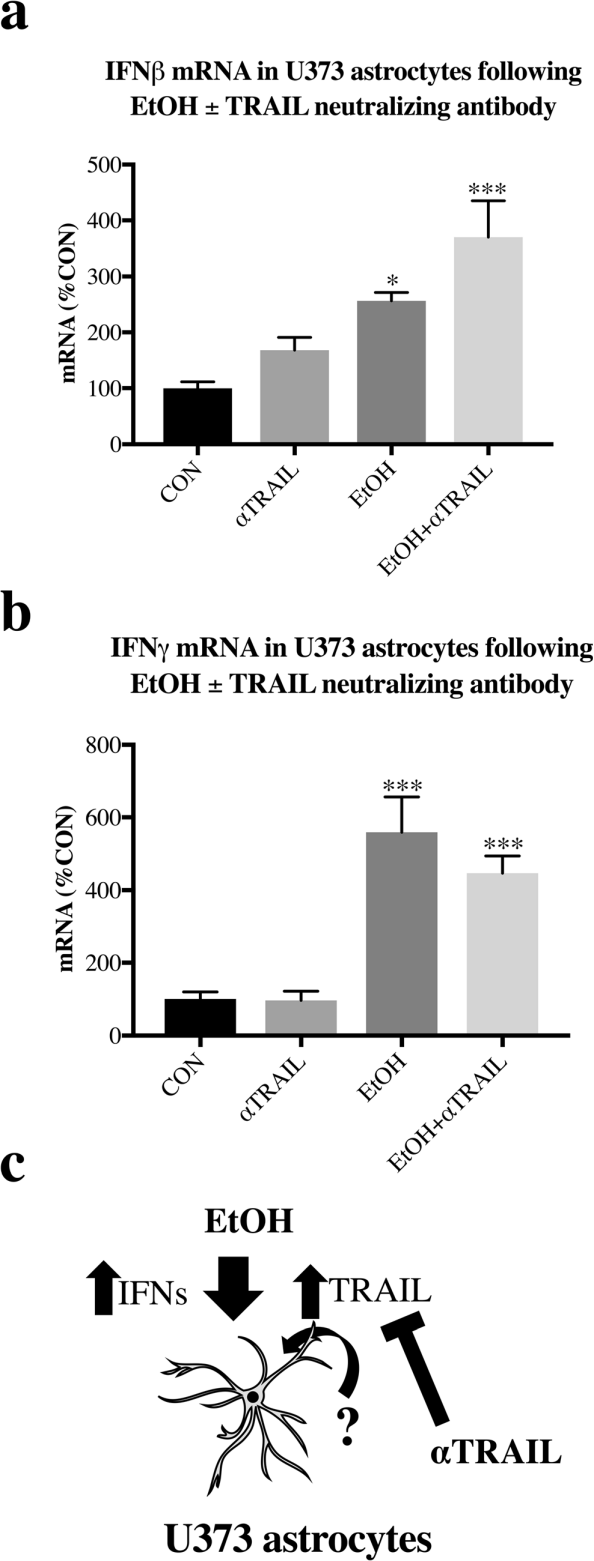
Ethanol induced interferon expression in U373 astrocytes is not blocked by TRAIL neutralizing antibody. U373 astrocytes were treated with EtOH (100 mM). Thirty minutes prior to EtOH, TRAIL neutralizing antibody (αTRAIL; 2 μg/mL) was added to the specified groups. Twenty-four hours later, cell lysates were collected for mRNA analysis using RT-PCR, with data represented as %CON (mean ± SEM). **a** IFNβ was increased by EtOH (257 ± 15%) which was not blocked by αTRAIL (370 ± 65%). One-way ANOVA [*F*(3,12) = 10.92, *p* = 0.001] followed by Dunnet’s post hoc. **b** IFNγ was increased by EtOH (559 ± 98%), which was not blocked by αTRAIL (447 ± 47%). One-way ANOVA [*F*(3,10) = 23.62, *p* < 0.0001] followed by Dunnet’s post hoc. **c** Schematic illustrating that EtOH increases TRAIL U373 astrocytes, and that TRAIL neutralization does not affect EtOH-induced IFN induction. *p < 0.05, ***p < 0.001 vs. CON

**Fig. 6:**
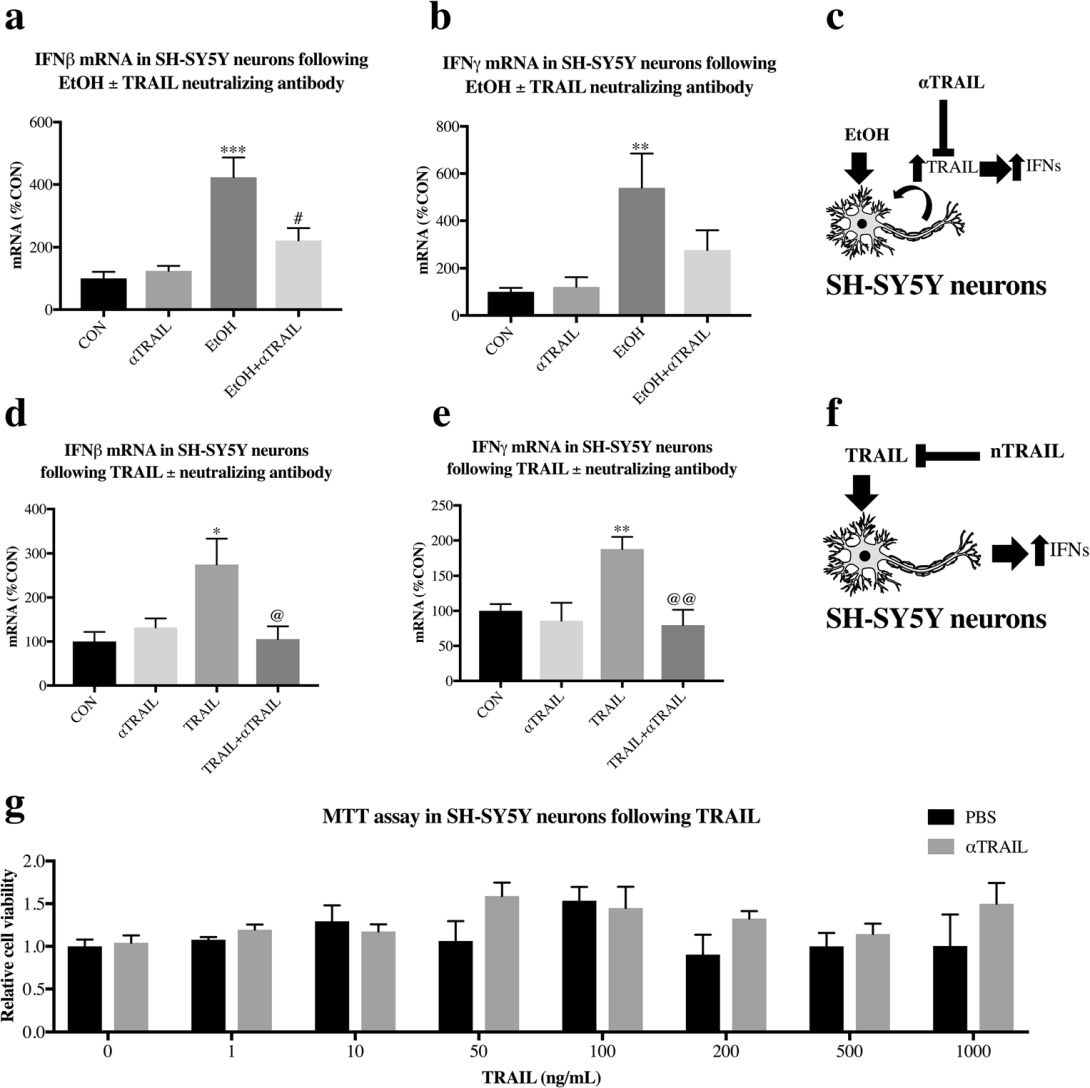
Ethanol and TRAIL-induced interferons is blocked by TRAIL neutralizing antibody in SH-SY5Y neurons. **a**–**c** SH-SY5Y neurons were treated with EtOH (100 mM). Thirty minutes prior to EtOH, TRAIL neutralizing antibody (αTRAIL; 2 μg/mL) was added to the specified groups. Twenty-four hours later, cell lysates were collected for mRNA analysis using RT-PCR, with data represented as %CON (mean ± SEM). **a** IFNβ was increased by EtOH (433 ± 64%), which was blocked by αTRAIL (221 ± 39%). One-way ANOVA [*F*(3,17) = 13.62, *p* < 0.0001] followed by Tukey’s post hoc. **b** IFNγ was increased by EtOH (539 ± 146%), which was blocked by αTRAIL (278 ± 82%). One-way ANOVA [*F*(3,17) = 5.83, *p* = 0.0063] followed by Tukey’s post hoc. **c** Schematic illustrating EtOH increases IFNs in SH-SY5Y neurons that is blocked by TRAIL neutralizing antibody. **d**–**f** SH-SY5Y neurons were treated with TRAIL (200 ng/mL). Thirty minutes prior to TRAIL, TRAIL neutralizing antibody (αTRAIL; 2 μg/mL) was added to the specified groups. Twenty-four hours later, cell lysates were collected for mRNA analysis using RT-PCR. **d** IFNβ was increased by TRAIL (275 ± 58%) and was blocked by αTRAIL (106 ± 29%). One-way ANOVA [*F*(3,16) = 5.31, *p* = 0.0099] followed by Tukey’s post hoc. **e** IFNγ was increased by TRAIL (188 ± 17%) and was blocked by αTRAIL (80 ± 22%). One-way ANOVA [*F*(3,12) = 5.92, *p* = 0.010] followed by Tukey’s post hoc. **f** Schematic illustrating that TRAIL increases IFNs in SH-SY5Y neurons that is blocked by TRAIL neutralizing antibody. **g** MTT assay conducted in SH-SY5Y neuron with varying TRAIL concentrations (0–1000 ng/mL), with either αTRAIL (2 μg/mL) or a volume-equivalent of vehicle (PBS). No significant loss of viability was observed at any TRAIL concentration. Two-way ANOVA, no main effect of TRAIL, [*F*(7,32) = 1.43, *p* = 0.22]. *p < 0.05, **p < 0.01, ***p < 0.001 vs. CON; #*p* < 0.05 vs. EtOH; @*p* < 0.05, @@*p* < 0.01 vs. TRAIL

Little is known about TRAIL signaling in brain, although in breast carcinoma cells (Kumar-Sinha et al. 2002), TRAIL induces IFNs. To determine if EtOH-induced TRAIL is linked to IFN induction, we used a TRAIL neutralizing antibody (αTRAIL) to block TRAIL. The TRAIL neutralizing antibody was added to the media 30 min prior to EtOH treatment of U373 astrocytes. While EtOH increased expression of IFNβ (2.6-fold, *p* < 0.05) and IFNγ (5.6-fold, *p* < 0.001), these increases were not blocked by the TRAIL neutralizing antibody (Fig. 5a–c). These data suggest that EtOH-induced IFN expression in astrocytes is not dependent on autocrine TRAIL signaling in U373 astrocytes and is more likely to be related to TLR3 or other EtOH-induced signaling. We found that both EtOH and TRAIL induced IFNs in SH-SY5Y neurons (Fig. 6a–f). Addition of the TRAIL neutralizing antibody 30 min prior to EtOH treatment of SH-SY5Y neurons did prevent EtOH-induced IFNβ and IFNγ gene induction (Fig. 6a–c). Similar to the effects of EtOH, the TRAIL neutralizing antibody blocked IFNβ and IFNγ induction in response to recombinant TRAIL (200 ng/mL) (Fig. 6d–f). Since TRAIL signaling has been shown to have neurodegenerative effects under certain conditions (Aktas et al. 2005; Cantarella et al. 2003), we examined whether TRAIL induces cell death in SH-SY5Y. TRAIL did not induce cell death at any of the concentrations examined (Fig. 6g). EtOH similarly does not induce cell death in SH-SY5Y neurons (Lawrimore and Crews 2017). Thus, these data suggest that EtOH induces IFN expression via TRAIL signaling in SH-SY5Y neurons in the absence of cell death.

**Fig. 7:**
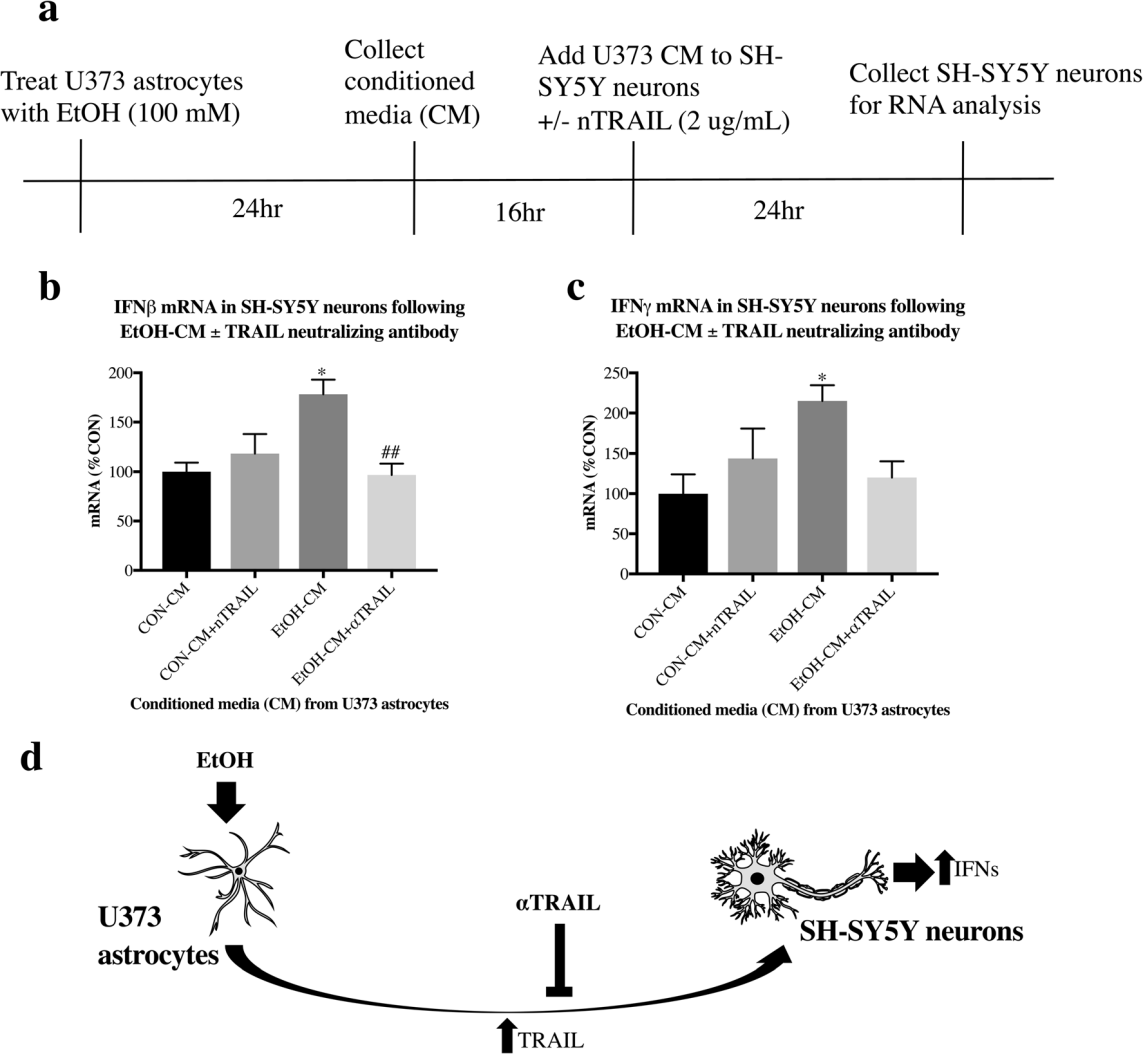
Ethanol-induced TRAIL from U373 astrocytes induces interferons in SH-SY5Y neurons. **a** Schematic diagraming treatment outline. U373 astrocytes were treated with EtOH (100 mM, 24 h) followed by media removal. EtOH was allowed to evaporate from the media before transferring to SH-SY5Y neurons. TRAIL neutralizing antibody (αTRAIL) was added immediately following media transfer to the specified groups. Twenty-four hours later, cell lysates were collected for mRNA analysis using RT-PCR. **b** IFNβ was increased by ethanol-conditioned media (EtOH-CM; 215 ± 19%), which was blocked by αTRAIL (120 ± 20%). One-way ANOVA [*F*(3,15) = 6.67, *p* = 0.0044] followed by Tukey’s post hoc. **c** IFNγ was increased by EtOH-CM (178 ± 15%), which was blocked by αTRAIL (97 ± 11%). One-way ANOVA [*F*(3,17) = 3.15, *p* = 0.0421] followed by Tukey’s post hoc. **d** Schematic outlining signaling pathway between EtOH treated U373 and induction of interferons in SH-SY5Y neurons. *p < 0.05 vs. CON; #*p* < 0.05 vs. EtOH-CM

Since astrocytes secrete TRAIL in response to EtOH, but do not show IFN responses in response to TRAIL, we hypothesized that neurons might respond to TRAIL from astrocytes in a paracrine fashion. We treated U373 astrocytes with EtOH for 24 h then collected the media. To examine EtOH-induced signaling molecules separate from the direct effects of EtOH, we allowed the EtOH to evaporate overnight (Walter and Crews 2017). U373-astrocyte-derived EtOH conditioned media (EtOH-CM) was collected and then transferred to SH-SY5Y neurons with or without the addition of the TRAIL neutralizing antibody. We found that EtOH-CM induced both IFNβ (2.2-fold, *p* < 0.05) and IFNγ (1.8-fold, *p* < 0.05) in SH-SY5Y neurons. Interestingly, this induction of IFN was abolished by the TRAIL neutralizing antibody (Fig. 7b, c). These data suggest that EtOH releases TRAIL from U373 human astrocytes that induce IFN expression in SH-SY5Y neurons. Thus, EtOH causes both autocrine and paracrine TRAIL-mediated IFN induction in neurons. EtOH can directly cause IFN induction in neurons. Further, astrocytes communicate with neurons via TRAIL release in response to EtOH, leading to IFN induction.

### EtOH and Poly(I:C) induce TRAIL in mouse brain

**Fig. 8:**
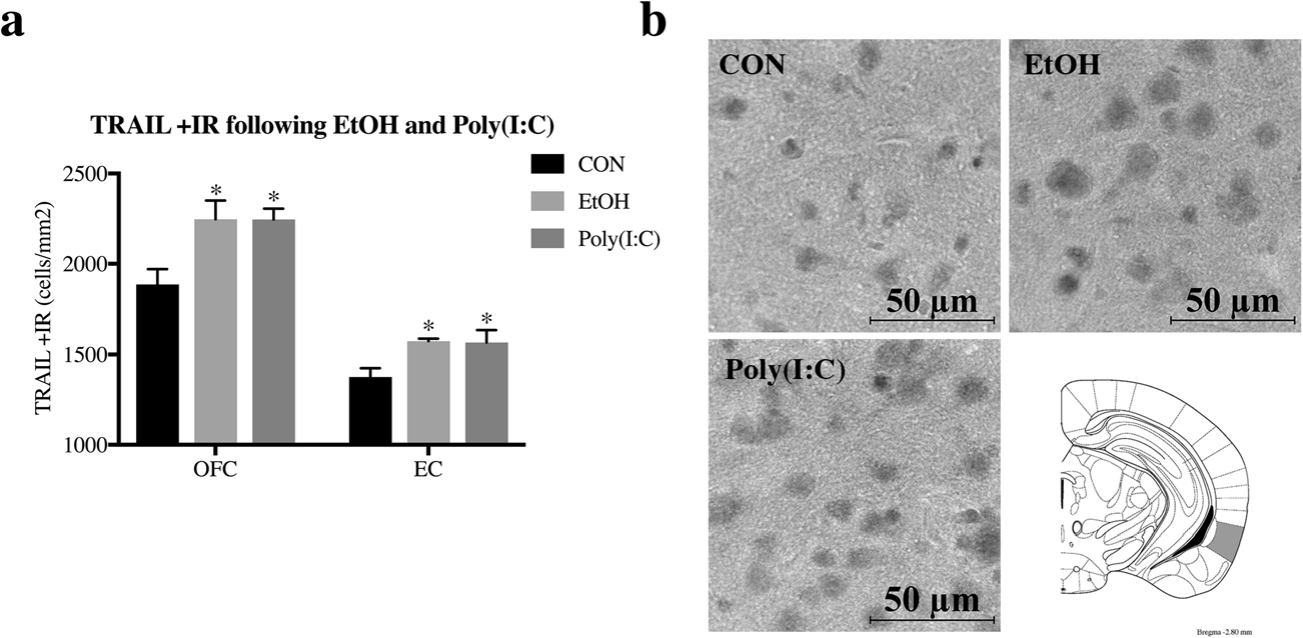
Ethanol and Poly(I:C) increase TRAIL +IR in mouse orbitofrontal and entorhinal cortex. C57BL6/J male mice (8 weeks old) were treated with either EtOH (once daily, 10 days, 5 g/kg, i.g.), Poly(I:C) (single dose, 13 mg/kg, i.p.), water (once daily, 10 days, i.g.), or saline (i.p.). Since no significant differences were seen between water (i.g.) and saline (i.p.) controls, these were grouped together into a single control [CON] group. Brains were processed for immunohistochemical staining by Neurosciences Associates and stained for TRAIL positive immunoreactive (+IR) cells. **a** The number of TRAIL +IR cells in both orbitofrontal and entorhinal cortex was increased by EtOH (*p* < 0.05) and Poly(I:C) (*p* < 0.05). One-way ANOVA [*F*(2,10) = 6.19, *p* = 0.0178] followed by Dunnet’s post hoc in orbitofrontal cortex, and one-way ANOVA [*F*(2,10)] = 4.22, *p* = 0.046] followed by Dunnet’s post hoc in entorhinal cortex. **b** Representative image of TRAIL +IR cells in entorhinal cortex, imaged at ×100. Positive cells are indicated by darker staining. *p < 0.05 vs. CON

To determine if EtOH and/or the TLR3 agonist Poly(I:C) alters brain TRAIL expression in vivo, we used a mouse model of chronic EtOH binge as well as a single acute Poly(I:C) treatment. C57BL/6 mice underwent a 10-day EtOH binge (5 g/kg, daily i.g.) or a single dose of TLR3 agonist Poly(I:C) (13 mg/kg, i.p.) and sacrificed 24 h later to assess TRAIL expression in brain We found both EtOH and Poly IC increased the number of TRAIL immunopositive reactive cells (TRAIL +IR) by approximately 20% in orbitofrontal cortex and entorhinal cortex relative to controls; EtOH (*p* < 0.05) or Poly(I:C) (*p* < 0.05) (Fig. 8a, b). We also observed that the TLR4 agonist LPS (0.5 mg/kg, i.p.) caused a similar induction of TRAIL (data not shown), suggesting TRAIL upregulation is sensitive to several immune stimuli. Thus, TRAIL is induced by EtOH and Poly(I:C) in mouse brain.

## Discussion

**Fig. 9:**
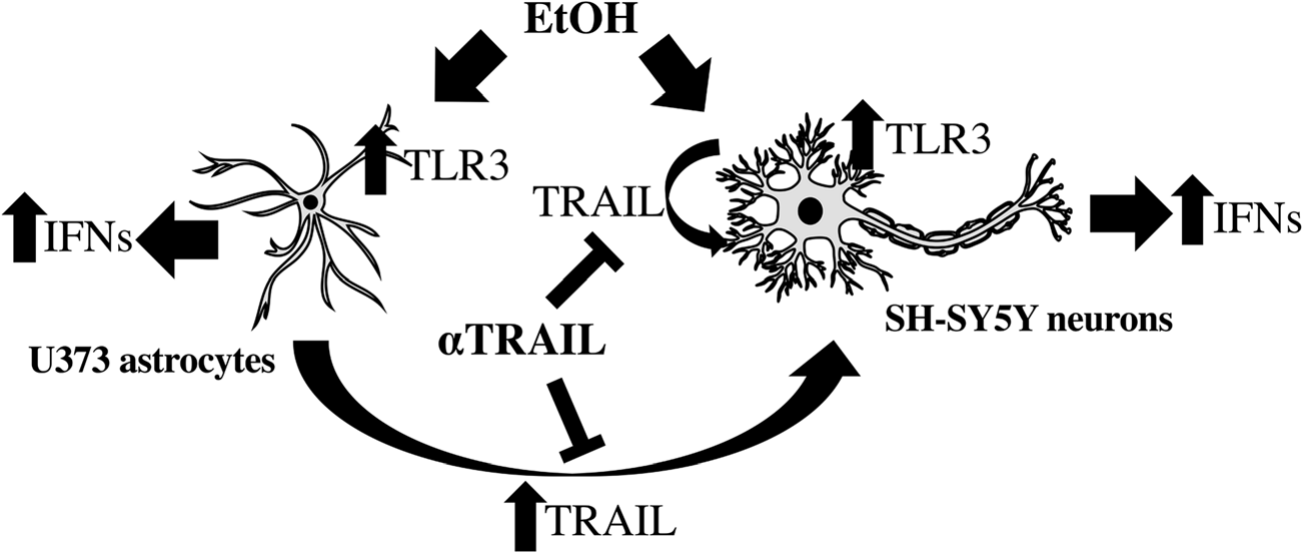
Summary of TRAIL-IFN interaction between U373 astrocytes and SH-SY5Y neurons. EtOH increases TLR3 and IFN mRNA expression in both U373 astrocytes and SH-SY5Y neurons. EtOH also increases release of TRAIL from U373 astrocytes, which further causes an increase of IFN expression in SH-SY5Y neurons. Blocking TRAIL signaling via a neutralizing antibody (αTRAIL) negates both EtOH-induced and TRAIL-induced IFN expression in SH-SY5Y neurons

In this manuscript, we found that EtOH induces TLR3, IFNs, and TRAIL in both U373 astrocytes and SH-SY5Y neurons, but not in BV2 microglia. Our studies find that TRAIL contributes to both autocrine and paracrine forms of neuroimmune signaling in brain, as diagramed in Fig. 9. In SH-SY5Y neurons, EtOH directly induced IFN gene expression that was prevented by TRAIL neutralizing antibody. Also, astrocytes signal in a paracrine fashion to neurons via TRAIL to induce neuronal IFNs. Several other studies have identified paracrine glial-neuronal signaling pathways in the brain, such as fractalkine which may modulate synaptic plasticity (Sheridan et al. 2014) as well as dysregulated amyloid β-complement signaling in Alzheimer’s disease models (Lian et al. 2015). Our study finds a novel paracrine signaling mechanism from astrocytes to neurons involving TRAIL-mediated IFN induction in neurons due to alcohol. The relationship between TRAIL and IFNs has been identified previously in other cell types (Huang et al. 2009; Peteranderl and Herold 2017). In peripheral immune cells, TRAIL is often considered an IRG, one of dozens of genes induced by IFN receptor signaling (Huang et al. 2009; Levy and Darnell Jr 2002; Peteranderl and Herold 2017; Sato et al. 2001). We found that TRAIL induces IFNs in SH-SY5Y neurons. This has also been observed previously in breast carcinoma cells, showing a reciprocal interaction between these signaling systems (Kumar-Sinha et al. 2002). TRAIL was originally discovered to be involved in the initiation of cell death pathways (Pan et al. 1997). However, TRAIL has also been found to result in cytokine induction through NFkB and IRF mediated pathways (Henry and Martin 2017; Kumar-Sinha et al. 2002).

In our studies, TRAIL did not cause detectable cell death in SH-SY5Y at concentrations of 200 ng/mL (24-h treatment). These findings are consistent with previous studies in SH-SY5Y neurons finding a 16-h treatment with TRAIL (500 ng/mL) did not result in cell death (Ruggeri et al. 2016). Some studies find that a longer 48-h treatment of TRAIL (100 ng/mL) reduces SH-SY5Y neuronal viability by approximately 30% (Cantarella et al. 2003) and induction of cell death by TRAIL in resected human brain tissue (Nitsch et al. 2000). Others have found that TRAIL induces cell death in SH-SY5Y neurons after NGF treatment (Ruggeri et al. 2016). This suggests that cell death pathways activated by TRAIL in neurons can vary and may be slower to initiate than IFN signaling in SH-SY5Y. Nevertheless, in our setting, we observe neuronal induction of IFNs by TRAIL in the absence of clear cell death. While implications of this in vivo are unclear, it is possible that persistent or ongoing upregulation of TRAIL signaling is needed to initiate neuronal cell death, as is observed in multiple sclerosis models (Aktas et al. 2005).

We found important differences between TRAIL-IFN signaling in response to EtOH between neurons and astrocytes. TRAIL antagonism prevented EtOH induction of IFNs in neurons but not astrocytes. Previously, we also found that TLR3 agonist Poly(I:C) induces a strong cytokine response in SH-SY5Y neurons (Lawrimore and Crews 2017); however, our current data suggests TRAIL, rather than TLR3 may be required for EtOH-induction of IFN in neurons. In astrocytes, however, we suspect that TLR3 signaling may be required for EtOH-induced IFN-TRAIL signaling. TLR3 stimulation via Poly(I:C) in U373 astrocytes strongly upregulated expression of IFNs as well TRAIL. TLR3 is upstream of IFN signaling and is prominently expressed in astrocytes (Farina et al. 2005; McCarthy et al. 2017a). We demonstrated here that Poly(I:C) strongly upregulates cytokines as well as IFNs in U373 astrocytes (see Table 4), consistent with studies conducted in primary human astrocytes (Farina et al. 2005; Serramia et al. 2015). The subdued effect of TLR4 and TLR7 stimulation in U373 astrocytes we observed is similar to previous studies that found little proinflammatory stimulation by TLR agonists other than Poly(I:C) in primary astrocytes (Farina et al. 2005). This is consistent with strong TLR3 responsivity in astrocytes. Importantly, we uniquely demonstrated that Poly(I:C) induces a strong upregulation of TRAIL as well in U373 astrocytes, with TRAIL antagonism having no effect on IFN induction, supporting the hypothesis that TLR3 is upstream of IFNs and TRAIL in astrocytes. Further, our finding of increased media TRAIL at 1-h post-treatment suggests that cleavage of membrane bound TRAIL might also be occurring, as TRAIL has been shown to be cleaved and released by cysteine proteases (Mariani and Krammer 1998). Future studies are required to definitively dissect the mechanism of TRAIL secretion in astrocytes. While we found that Poly(I:C) induces TRAIL in U373 astrocytes, previous studies indicate that Poly(I:C) also upregulates other IFN stimulated genes in astrocytes, such as ISG54 and ISG56 (Imaizumi et al. 2014), suggesting that further research into TLR3-TRAIL-IFN-mediated responses in astrocytes is warranted, especially given the ability of astrocytes to secrete factors that alter neurons (Liddelow et al. 2017).

While in the periphery IFNs are known for their anti-viral activity, in the brain, they may play different roles. In particular, exogenous IFNs have been well-documented in their ability to cause depression in clinical settings and in rodents (Borsini et al. 2017; Callaghan et al. 2018; Fritz et al. 2018; Lian et al. 2015; Mina et al. 2015; Pinto and Andrade 2016). Negative affect and depressive phenotypes are critical components in the cycle of addiction (Coleman Jr. and Crews 2018; Koob 2015; Koob and Volkow 2010; Volkow et al. 2016). Both the negative affect/depressive phenotypes found with exogenous IFNs and the development of negative affect in rodent models of alcohol addiction follow similar patterns of progressive onset (Breese et al. 2008; Breese et al. 2005; Raison et al. 2009; Wills et al. 2009; Zheng et al. 2014). Further, IFNγ was found to be upregulated in postmortem human alcoholic cortex (Johnson et al. 2015), and chronic EtOH in vivo can induce IFNγ (Duncan et al. 2016; Pascual et al. 2015). IFNγ has also been suggested to play a role in modulating social behavior in mice (Filiano et al. 2016), and IFNγ knockout mice lack LPS-induced conditioned place aversion, indicating a role for IFNγ in negative affect behaviors (Fritz et al. 2018). Therefore, blocking IFN induction by TRAIL in neurons could be a potential cell-specific target for AUDs and other neurologic disorders (Cantarella et al. 2015). Further research is needed to investigate the possible therapeutic potential of TRAIL inhibition as well as the mechanisms underlying cell-to-cell TRAIL signaling in the brain.

Although microglia are generally associated with neuroimmune signaling and primary microglia have been reported to release IFNβ (McDonough et al. 2017), we find IFN and TLR3 receptor induction by EtOH is primarily in astrocytes and neurons. Astrocytes play a vital role in modulating neuronal plasticity in the brain (Haydon and Nedergaard 2014) and are becoming a prominent focus of alcohol research (Adermark and Bowers 2016). Previous studies have indicated that the astrocyte secretome, such as multiple extracellular matrix proteins, is changed following chronic EtOH exposure during early postnatal life (Trindade et al. 2016), and that EtOH upregulates immune associated factors (e.g., COX-2, iNOS) in primary cultured rodent astrocytes (Valles et al. 2004). Thus, though microglia undoubtedly play important roles in neuroimmune responses to EtOH, the contribution of astrocytes should be further investigated.

It is important to note that our in vitro studies use immortalized cell lines, which may have different responses compared to primary cells or in vivo. However, these selected cell lines are widely utilized cell culture models for providing insight into neuronal, microglial, and astrocyte biology, including immune function. For instance, retinoic acid-differentiated SH-SY5Y neurons have been used extensively in Parkinson’s disease models (for reviews, see Xicoy et al. 2017; Xie et al. 2010) and much like dopaminergic neurons in vivo can actively uptake MPP(+) (Korecka et al. 2013). SH-SY5Y also have increased expression of immediate early genes (FOS and ARC) following stimulation with KCl, a response indicative of neuronal activation (Barry et al. 2017). SH-SY5Y also model innate immune signaling similar to primary neurons, as in our previous study, we found that TLR2, TLR3, TLR4, TLR7, and TLR8 are expressed in SH-SY5Y (Lawrimore and Crews 2017), similar to studies using primary neurons in mice and in vivo (Lehmann et al. 2012; Lok et al. 2015; Tang et al. 2007). BV2 microglia demonstrate a similar response to primary microglia following LPS (TLR4) stimulation (Henn et al. 2009). U373 express astrocyte markers and have similar cytokine responses as primary astrocytes (Imaizumi et al. 2013; Rosenberger et al. 2016). Further, similar to our findings in U373 astrocytes, primary human astrocytes have also been found to express IFN response gene TRAIL following treatment with proinflammatory cytokines (Choi et al. 1999). Therefore, though cell line experiments must be evaluated in the proper context, the literature indicates that the cell lines we have selected are appropriate in investigating innate immune function.

We also extended our in vitro findings with our in vivo experiments. To investigate the impact of EtOH on TRAIL in vivo in brain, we used our 10-day binge model of EtOH intoxication in mice (Qin and Crews 2012; Qin et al. 2008) that induces brain neuroimmune gene responses and determined TRAIL +IHC. TRAIL +IR was increased in both orbitofrontal and entorhinal cortex by EtOH treatment, regions that are affected by addiction pathology (Crews and Boettiger 2009; Schoenbaum and Shaham 2008). Thus, EtOH induces TRAIL in our selected in vitro models as well as in our in vivo binge EtOH model of AUD induced cortical pathology. In conclusion, we identify a novel signaling system whereby EtOH induces IFNs in neurons via neuronal and astrocytic secretion of TRAIL.
